# Single-Molecule Real-Time and Illumina Sequencing to Analyze Transcriptional Regulation of Flavonoid Synthesis in Blueberry

**DOI:** 10.3389/fpls.2021.754325

**Published:** 2021-09-30

**Authors:** Qi Tang, Fu-Mei Chi, Hong-Di Liu, Hong-Jun Zhang, Yang Song

**Affiliations:** Key Laboratory of Biology and Genetic Improvement of Horticultural Crops (Germplasm Resources Utilization), Research Institute of Pomology, Chinese Academy of Agricultural Sciences, Ministry of Agriculture, Xingcheng, China

**Keywords:** transcriptome, fruit development, SMRT sequencing, RNA-seq, flavonoid biosynthesis, R2R3 MYB, *Vaccinium corymbosum*

## Abstract

Blueberries (*Vaccinium corymbosum*) contain large amounts of flavonoids, which play important roles in the plant’s ability to resist stress and can also have beneficial effects on human health when the fruits are eaten. However, the molecular mechanisms that regulate flavonoid synthesis in blueberries are still unclear. In this study, we combined two different transcriptome sequencing platforms, single-molecule real-time (SMRT) and Illumina sequencing, to elucidate the flavonoid synthetic pathways in blueberries. We analyzed transcript quantity, length, and the number of annotated genes. We mined genes associated with flavonoid synthesis (such as anthocyanins, flavonols, and proanthocyanidins) and employed fluorescence quantitative PCR to analyze the expression of these genes and their correlation with flavonoid synthesis. We discovered one R2R3 MYB transcription factor from the sequencing library, *VcMYB1*, that can positively regulate anthocyanin synthesis in blueberries. *VcMYB1* is mainly expressed in colored (mature) fruits. Experiments showed that overexpression and transient expression of *VcMYB1* promoted anthocyanin synthesis in *Arabidopsis*, tobacco (*Nicotiana benthamiana*) plants and green blueberry fruits. Yeast one-hybrid (Y1H) assay, electrophoretic mobility shift assay, and transient expression experiments showed that VcMYB1 binds to the MYB binding site on the promoter of the structural gene for anthocyanin synthesis, VcMYB1 to positively regulate the transcription of *VcDFR*, thereby promoting anthocyanin synthesis. We also performed an in-depth investigation of transcriptional regulation of anthocyanin synthesis. This study provides background information and data for studying the synthetic pathways of flavonoids and other secondary metabolites in blueberries.

## Introduction

Blueberries (*Vaccinium corymbosum*, Ericaceae) are one of the most popular fruits globally. In North America, blueberries are grown along the Atlantic coast of the United States and in southeastern Canada. In China, blueberries are cultivated in more than 27 provinces, including Shandong, Liaoning, and Guizhou. By the end of 2020, China’s blueberry cultivation area had reached 66,400 hm^2^, and total production was 347,200 tons (Li et al., 2021). Thus, blueberries are becoming one of the most important fruit crops in China.

Blueberries contain large amounts of anthocyanins, proanthocyanidins, and flavonols, which are the three most common flavonoids. These flavonoids play important roles in fruit coloring, they span from functions in regulating plant development, pigmentation, to an array of roles in defense and signaling between plants and microorganisms, and flavonoid-rich mixed berries maintain and improve cognitive function over a 6 h period in young healthy adults (Ulrike, 2018; Adrian et al., 2019). Anthocyanin is the most common flavonoid and is responsible for the blue and red colors of the fruit. Therefore, elucidating the molecular mechanisms of anthocyanin synthesis would be valuable as a way to develop tools for modifying fruit quality and specifically enriching the formation of fruit color.

Usually MBW protein complexes, consisting of R2R3 MYB, basic helix-loop-helix (bHLH), and WD-repeat proteins regulate the synthesis of plant flavonoids and this has been verified in many plants. The R2R3 MYB transcription factor plays an important role in this protein complex. The plant R2R3 MYB gene family is extremely huge and their functions in other plants were also extensively studied. In apples, *MdMYB1* and *MdMYB10* can regulate the synthesis of anthocyanins (An et al., 2017a, An et al., 2020b). *PyMYB114* and *PybHLH3* can regulate anthocyanin synthesis in peaches (Yao et al., 2017). In bilberry (*Vaccinium myrtillus* L.), Wu C. et al. (2021) describe the complex MYBA locus and identify the key regulating MYB genes that determine anthocyanin production. In American cranberry (*Vaccinium macrocarpon* Ait.), the subgroup-6 (SG-6 or sg6) R2R3 MYB transcription factors likely act as anthocyanin biosynthesis regulators (Diaz et al., 2021). In blueberries, *VcMYBPA* can regulate proanthocyanidin synthesis (Zifkin et al., 2012). However, there are currently no studies on the R2R3 MYB gene that regulates anthocyanin synthesis in blueberries.

Most studies on flavonoid synthesis have been in model plants such as apples and peaches, whereas fewer studies have used blueberries (Zhou et al., 2015; An et al., 2017a; An et al., 2020b). One reason that little is known about the genetic mechanisms regulating flavonoid production in blueberries is that these plants are polyploid and have high heterozygosity; thus, genome information and analyses of gene function in blueberry has lagged behind other species (Costich et al., 1993; Rowland et al., 2012). In recent years, with the development of high-throughput sequencing technologies, transcriptome sequencing has become an important method for studying the regulation of gene expression. Transcriptome sequencing can be used to rapidly obtain almost all transcript information of a specific tissue or organ under certain conditions. The use of RNA sequencing (RNA-seq) combined with bioinformatics has provided new research ideas and routes for transcriptomics research. This combined technique is particularly suited for species for which genomic data are not yet available and has been used widely to perform studies on the formation of quality fruits and the mechanisms of stress resistance in many species, such as peaches (Choi et al., 2021), grapes (Xu et al., 2021), sweet oranges (Yao et al., 2020), bananas (Kaushal et al., 2021), and rice (Bakade et al., 2021).

Some studies have employed transcriptome sequencing (Illumina sequencing) technologies and expressed sequence tag (EST) library methods to analyze the metabolic mechanisms (Liang et al., 2020; Wu P. et al., 2021). Jaakola et al. (2002) and Zifkin et al. (2012) analyzed the correlation between expression of genes related to flavonoid synthesis at different developmental stages and flavonoid content in highbush blueberries (*V. corymbosum* “Rubel”) and wild blueberries (*Vaccinium myrtillus*), respectively. Li Y. Y. et al. (2012) and Sun et al. (2015) both employed *de novo* transcriptome sequencing to analyze the differential expression of genes related to flavonoid synthesis in red and white fruits of cranberries (*V. macrocarpon* “Bergman”) and in mature blueberry skins and pulp (*V. corymbosum* “Northland”), respectively. Li et al. (2016) also employed *de novo* transcriptome sequencing to analyze the expression of structural genes related to anthocyanin biosynthesis during different color stages of blueberries (*V. corymbosum* “Sierra”). Still, there is less genomic, transcriptomic, and proteomic molecular data on blueberries compared with other species. In addition, Although Illumina sequencing technology has high accuracy, due to the limitation of reading length, the assembled transcripts are incomplete and the accuracy is low (Xu et al., 2015; Li et al., 2017). As an alternative, PacBio single-molecule real-time sequencing (SMRT) technology can be used to construct libraries with different inserts and has an advantage of ultra-long read lengths (5–8 kb). SMRT technology has been widely applied to multiple species (Minoche et al., 2015; Du et al., 2021). SMRT has also been applied in combination with Illumina sequencing in many studies (Xu et al., 2015; Li et al., 2017; Qian et al., 2020). Here, we aimed to combine SMRT and Illumina sequencing technologies to study transcriptome data in different developmental stages of blueberry (*V. corymbosum* “Duke”) to identify the genes that regulate flavonoid synthesis. “Duke” was selected because it is one of the main cultivated blueberry varieties in Shandong and Liaoning provinces in China. In addition, this cultivar has relatively higher flavonoid content and antioxidant activity (Prior et al., 1998).

This work will provide a platform for future research on improving fruit coloring and understanding the regulatory mechanisms for flavonoid synthesis in blueberries.

## Materials and Methods

### Plant Materials

The experiments were carried out at the College of Horticulture Sciences, Shandong Agricultural University (Tai’an, Shandong, China) and Ministry of Agriculture Key Laboratory for utilization of horticultural crop germplasm resources, Fruit Tree Research Institute, Chinese Academy of Agricultural Sciences (Xingcheng, Liaoning, China) from April 2018 to January 2021. The experimental material were 9-year-old blueberry (*V. corymbosum* “Duke”) seedlings that were collected from the small berry garden in the Fruit Tree Research Institute, Chinese Academy of Agricultural Sciences. Fruits were collected 40 days (green fruits), 65 days (pink fruits), or 80 days (blue fruits) after flowering. To ensure consistency across the materials, fruits were collected from the tips of inflorescences (first to mature), snap-frozen in liquid nitrogen, and stored at –80°C until analysis.

### Library Preparation and Single-Molecule Real-Time Sequencing

Plant total RNA extraction kits (TaKaRa, Beijing, China, No. 9769S) were used for extraction of total RNA from fruits of each of the three stages. Agarose gel electrophoresis (Agilent 2100 bioanalyzer, Agilent),^1^ and spectrophotometry (NanoDrop spectrophotometer, Thermo Fisher Scientific)^2^ were used to examine the integrity, purity, and quality of total RNA. Oligo (dT) magnetic beads were used for mRNA enrichment and the SMART PCR cDNA Synthesis Kit was used to synthesize full-length cDNA from mRNA. BluePippin (Sage Science)^3^ was used to screen full-length cDNA fragments and three types of insert libraries were constructed (1–2, 2–3, and 3–6 kb). PCR amplification was carried out on full-length, screened cDNA. End-terminal repair was carried out on full-length cDNA and the cDNAs were ligated to SMRT bell adapters before digestion using exonucleases. Secondary screening of the libraries was carried out using BluePippin to obtain the sequencing libraries. After library construction, Qubit 2.0 (Thermo Fisher Scientific) was used for quantitative analysis. The Agilent 2100 bioanalyzer (Agilent 2100 bioanalyzer) was used for examination of the insert fragments in the libraries. After the libraries passed the quality check, the PacBio RS II platform was used for full-length transcriptome sequencing. Two SMRT cells were used for sequencing the 1–2 and 3–6 kb fragment libraries, and three SMRT cells were used for sequencing the 2–3 kb fragment libraries. The LoRDEC software was used to correct the sequencing errors in the consensus transcripts using Illumina reads as the reference (Salmela and Rivals, 2014).

### Library Preparation and Illumina Sequencing

The methods for total RNA extraction from fruits and examination of total RNA integrity, purity, and quality were carried out as described above. After DNAse I digestion of total RNA from fruit samples, oligo (dT) magnetic beads were used for mRNA enrichment. After mRNAs were made into short fragments, first-strand cDNA and second-strand cDNA were synthesized, purified and recovered, and the sticky ends were repaired. An “A” adapter was added to the 3′ ends of the cDNA before selection of fragment sizes. Finally, PCR amplification was used to construct the libraries. Qubit 2.0 (Thermo Fisher Scientific; see text footnote 2) and Agilent 2100 bioanalyzers were used for examination of library concentration and the fragment sizes of the inserts in the constructed libraries. We used Quantitative PCR to verify quantitation of the effective concentrations of the libraries. After the libraries passed the quality check, the Illumina HiSeq 2500 sequencer was used for sequencing and the sequencing read length was 125 bp paired-ends. RNA samples from green, pink, and blue fruits were sequenced in duplicates.

### Sequence Data Analysis

The IsoSeq^TM^ protocol (Pacific Biosciences, United States) was used to process the sequenced reads using circular consensus sequencing RNA BIOLOGY 5. Effective subreads were obtained using the P_Fetch and P_Filter function (parameters: miniLength = 50, read Score = 0.75, artifact = –1,000) in the SMRT Analysis software suite.^4^ CCS was obtained from the P_CCS module using the parameter Min Complete Passes = 2 and Min Predicted Accuracy = 0. After examining for poly (A) signal and 5′ and 3′ adaptors, only the CCS with all three signals was considered as a FLNC read (Du et al., 2021). Unmerged subreads were also examined for the three signals, and those with three signals were incorporated into the final FLNC read set.

### Molecular Cloning of VcMYB1

Blueberry transcriptome data from Illumina and SMRT sequencing were used for alignment with COG, GO, and NR databases to obtain VcMYB genes. Phylogenetic analysis of all the excavated VcMYB protein sequences and *Arabidopsis* MYB protein sequences showed that only one VcMYB sequence has extremely high homology with the *Arabidopsis* sg6 R2R3 MYB protein, and it was named VcMYB1.

### qRT-PCR Analysis

Three RNA samples were isolated from fruits of each of the three fruit development stages of blueberries. RNA samples were isolated from leaves of *Arabidopsis*. Single-stranded cDNA was obtained using a reverse transcription kit (TaKaRa, Shiga, Japan). All qRT-PCR analyses were performed with three biological repeats and three techincal repeats. *Arabidopsis polyubiquitin* 10 (AtUBQ10) was used as housekeeping gene. Supplementary Table 4 shows the primers used.

### High Performance Liquid Chromatography Analysis of Flavonoid Content

The extraction methods for anthocyanins, flavonols, and proanthocyanidins were referenced from Prior et al. (2001); Lätti et al. (2010), and Pertuzatti et al. (2016), respectively. High performance liquid chromatography (HPLC) separation, identification and quantitation of anthocyanins, proanthocyanidins, and flavonols were performed on an Agilent 1100 Series system (Agilent, Germany), equipped with DAD (G1315B) and a LC/MSD Trap VL (G2445C VL) electrospray ionization mass spectrometry (ESIMSn) system that was coupled to an Agilent ChemStation (version B.01.03) data-processing station. The mass spectra data were processed with the Agilent LC/MS Trap software (Version 5.3).

ESI-MSn was used to identify the anthocyanins, proanthocyanidins, and flavonols profile and the following parameters were employed: positive ionization mode; dry gas, N2, 11 mL/min; drying temperature, 350°C; nebulizer, 65 psi; capillary, –2500 V; capillary exit offset, 70 V; skimmer1, 20 V; skimmer 2, 6 V; compound stability, 100%; scan range, 50–1,200 m/z. For the purposes of quantitation, For quantification, DAD chromatograms were extracted at 520 nm for anthocyanins, proanthocyanidins at 280 nm, and flavonols at 360 nm.

### Amino-Acid Sequence Analysis and Phylogenetic Tree Construction

The Protein BLAST program^5^ was used to obtain homologs of *Arabidopsis, Vitis vinifera* and *Malus domestica* MYB. The amino-acid secondary structure of VcMYB was predicted using the Simple Modular Architecture Research Tool (SMART) software program.^6^

### Ectopic Expression of VcMYB1 in Arabidopsis

The ORF (Open Reading Frame) was cloned and inserted into the pRI101-AN vector, and used to transform *Agrobacterium tumefaciens* GV3101. *Arabidopsis* plants were transformed with Agrobacterium by the floral dipping method. T1 VcMYB1 transgenic plants were selected on Murashige and Skoog (MS) media containing 50 mg L^–1^ kanamycin. Kanamycin-resistant T1 seedlings were transferred to soil and grown at 22°C in a growth chamber with a 16 h day length. T2 seedlings were selected on MS media containing 50 mg L^–1^ kanamycin.

### Induction of Anthocyanins by Transient Transformation of Tobacco

The Agrobacterium GV3101 strains containing the pRI101-VcMYB1 were infiltrated into the abaxial leaf surface of tobacco according to Espley et al. (2007). Each infiltration was performed using three leaves from the same plants. Photographs were taken 4–7 days after infiltration. To control for leaf-to-leaf variability, at least two plants were used for infiltration, and each leaf included negative controls (Agrobacterium with pRI101 empty vector).

### Blueberry Injection Assays

Fruit injection assays were carried out according to Li X. Y. et al. (2012), Li Y. Y. et al. (2012), and An et al. (2017b). The VcMYB1-pGreenII62-SK vector was generated by cloning the ORF of *VcMYB1* into the pGreenII62-SK vector. The mixed vectors and the *A. tumefaciens* solutions were injected into the fruit peels.

### Genome Walking Assays and Promoter Cloning

Chromosome walking techniques were used to isolate the promoter sequence for *VcDFR* and the method was referenced from the Genome Walking Kit (TaKaRa).^7^ Specific primers (SP1, SP2, and SP3) were designed according to the open reading frame of *VcDFR*. Specific primers and four degenerate primers in the reagent kit were used for three rounds of thermal asymmetric PCR. Finally, a 834 bp fragment was obtained through three rounds of PCR.

### Yeast One-Hybrid Assays

Y1H assays were performed with the yeast strain Y1HGold (Clontech).^8^ The full-length cDNA of *VcMYB1* was cloned and inserted into the pGADT7 vector. The promoter fragments of *VcDFR* were cloned into the pAbAi vector to generated the construct pAbAi-VcDFR_*pro*_, and then transformed into Y1HGold using a PEG/LiAc method. Yeast cells were cultured on the medium lacking Ura (SD/-Ura). Yeast colony PCR for confirming that positive plasmid has integrated into the Y1HGold genome, and then transformed pGADT7-VcMYB1 into Y1HGold-pAbAi-VcDFR_*pro*_. Yeast cells were cultured on media lacking Leu (SD/-Leu) with Aureobasidin A to identify possible interactions.

### Electrophoretic Mobility Shift Assay

The EMSA was performed using the LightShift Chemiluminescent EMSA Kit (Thermo Fisher Scientific, Waltham, MA, United States). Briefly, biotin-labeled probes were incubated in a 1 × binding buffer containing 5 mM MgCl_2_, 50 mM KCl and 2.5% glycerol with or without VcMYB1-His fusion proteins at room temperature for 20 min. An unlabeled probe was added to the reactions for unlabeled probe competition.

### Firefly Luciferase Complementation Assay

Transient expression assay in tobacco leaves were performed according to Shang et al. (2010). The VcDFR promoter was amplified and cloned into pGreenII0800-LUC vector, which generated the reporter constructs *VcDFR_*pro*_:Luc*. The effector (*35S_*pro*_:VcMYB1*) was constructed by cloning the ORF of *VcMYB1* into the pGreenII62-SK vector. Transformed leaves were sprayed with 100 mM luciferin, after which they were placed in darkness for 5 min before examining for luminescence. A charge-coupled device-imaging apparatus (NightOWL LB983 in conjunction with Indigo software) was used to collect the LUC images.

### Analysis of β-Glucuronidase Activity

The Agrobacterium GV3101 strains containing the pRI101-VcMYB1 generated effector constructs. Reporter constructs were generated using the promoter sequences of *VcDFR* cloned upstream of the β-glucuronidase (GUS) reporter gene in the pCAMBIA1301 vector, and used to transform Agrobacterium GV3101. For the transient expression assay, Agrobacterium GV3101 strains containing the pRI101-VcMYB1 were co-infiltrated into the abaxial leaf surface of tobacco. Each infection was performed in three biological replicates. After grow in a growth chamber for 3–4 days, the infected leaves were used to analyze GUS activity. Proteins were extracted from infected leaves and fluorescence was measured using a fluorometer (VersaFluor Fluorometer, Bio-Rad)^9^ as performed according to Jefferson et al. (1987). The values of GUS activity were calculated from three biological replicates.

## Results

### Sequencing Data Analysis

**FIGURE 1:**
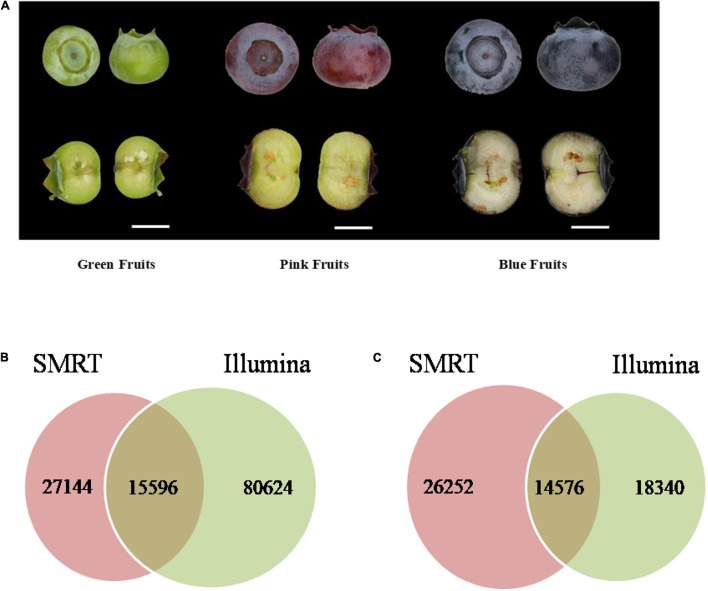
Blueberry (Vaccinium corymbosum “Duke”) fruits of different developmental stages were used in molecular and chemical analyses and Statistical analysis of sequencing data. **(A)** Green (40 days since flowering), pink (65 days), and blue (80 days) fruits were used in the analyses. Scale bar = 7 mm. Venn diagrams of **(B)** the number of common and unique sequences and **(C)** the number of common and unique annotated genes obtained in Illumina and SMRT sequencing.

The Illumina and SMRT sequencing platforms were used to analyze transcriptome data of blueberries. First, the Hiseq 2500 platform was used for sequencing of three samples from blueberry fruits of each of three developmental stage, green (diameter 8–10 mm), pink (12–14 mm), and blue (12–14 mm) (Figure 1A). Samples were sequenced in duplicates. After removal of sequencing adapters and primer sequences the high-quality reads (clean data) totaled 112,625,479 (Table 1) and the total number of bases was 28,376,811,360. After Trinity assembly, a total of 104,068 Unigenes were obtained (Supplementary Table 1).

**TABLE 1:** Illumina and SMRT sequencing data.

**Sample ID**	**Sequencing ID**	**Hiseq2500 reads**	**SMRT library**	**1–2 kb**	**2–3 kb**	**3–6 kb**	**Total**
Green fruits-1	Berry-1	19,386,127	No. of SMRT cells	2	3	2	7
Green fruits-2	Berry-2	16,543,686	Subreads (post-filter)	165,107	241,041	166,347	572,495
Pink fruits -1	Berry-3	21,144,486	reads of insert	136,466	189,732	132,766	458,964
Pink fruits -2	Berry-4	19,236,851	Mean read of insert length	1,985	3,053	3,776	2,938
Blue fruits-1	Berry-5	18,439,689	Mean read quality of insert	93.60%	90.43%	90.62	91.55%
Blue fruits-2	Berry-6	17,874,640	Mean number of passes	6	4	3	
	Total	112,625,479	Number of full-length non-chimeric reads (FLNC)	66,289	85,628	57,798	209,715
			Average FLNC read length	1,757	2,823	3,775	

Next, SMRT sequencing was carried out on the same samples. Equal amounts of six types of mRNAs from the three fruit stages were mixed to construct a sample pool. Three insert libraries were constructed and seven SMRT cell (two cells for 1–2 kb fragments, three cells for 2–3 kb fragments, and two cells for 3–6 kb fragments) sequencing microarrays were used. After removal of adapters and low-quality reads, 572,495 subreads (1.9 billion) were obtained. These subreads were screened to obtain 458,964 reads of insert (RoIs), of which there were 209,715 full-length non-chimeric reads (FLNCs) with 5′ and 3′ primer sequences and poly (A) sequences (Table 1). The average length of RoIs and FLNC reads were 2,938 and 2,785 bp, respectively. The iterative isoform-clustering (ICE) algorithm and Quiver software were used for sequence calibration and sequences with accuracy greater than 0.99 were retained to obtain 117,784 consensus reads. Then, CD-HIT EST software was used to remove redundant sequences from the above high-quality transcripts. We obtained 42,740 non-redundant sequences with an average length of 2,452 bases (Supplementary Table 1).

Although Illumina sequencing has extremely high coverage, the transcripts assembled by Trinity cannot represent full-length cDNA. In this study, the Illumina sequencing results of blueberry messenger RNA (mRNA) samples showed that approximately 37.81% of Unigenes were shorter than 300 bases. The number of Unigenes that were greater than 1, 2, and 3 kb were 18,849, 7,042, and 2,476, respectively, with an average length of 677 bases. In the SMRT sequencing data, the percentage of RoIs that were smaller than 300 bases was only 5.6%. The number of FLNCs that were greater than 1, 2, and 3 kb were 42,646, 24,312, and 9,896, respectively. The average length of FLNC reads in the three insert libraries (1–2, 2–3, and 3–6 kb) were 1,757, 2,823, and 3,775 bases. Overall, sequences obtained through SMRT sequencing were longer and the transcripts were more intact.

Blast alignment analysis of the corresponding relationship between 104,068 Unigene sequences obtained from Illumina sequencing and 42,740 non-redundant sequences from SMRT sequencing showed that the sequences do not show one-to-one correspondence as a single SMRT transcript corresponds to a single or multiple Illumina transcripts. There were 15,596 transcripts present in both Illumina and SMRT sequencing; 27,144 transcripts were only found in SMRT sequencing, and 80,624 transcripts were only found in Illumina sequencing (Figure 1B). The Unigene sequences obtained from Illumina sequencing and the non-redundant sequences obtained from SMRT sequencing were used for data alignment in Clusters of Orthologous Groups (COG), Gene Ontology (GO), and non-redundant (NR) databases. The number of annotated genes obtained through Illumina sequencing was lower than that obtained through SMRT sequencing. There were 32,916 annotated genes that were obtained through Illumina sequencing, which accounted for 31.6% of Unigenes and there were 40,828 annotated genes that were obtained through SMRT sequencing, which accounted for 95.5% of non-redundant sequences. Of the obtained annotated genes, 14,576 genes were common to both Illumina and SMRT sequencing; 18,340 genes were only found in Illumina sequencing, and 26,252, genes were only found in SMRT sequencing (Figure 1C).

We used three methods [coding-non-coding index (CNCI), coding potential calculator (CPC), and Pfamscan (PFAM)] to predict long non-coding RNAs (lncRNAs) longer than 200 bases. We obtained 6,251, 3,322, and 7,610 potential lncRNA sequences, respectively, using these three methods. Among these sequences, 2,171 potential lncRNA sequences simultaneously appeared in all three methods and these sequences were regarded as candidate lncRNAs in the target dataset (Supplementary Figure 1).

### Analysis of the Expression of Genes Associated With Flavonoid Synthesis and Accumulated Flavonoids

Plants employ the phenylalanine metabolic pathway to synthesize flavonoids such as anthocyanins, proanthocyanidins, and flavonols. Here, blueberry transcriptome data from Illumina and SMRT sequencing were used for alignment with COG, GO, and NR databases to obtain genes that regulate flavonoid synthesis.

**FIGURE 2:**
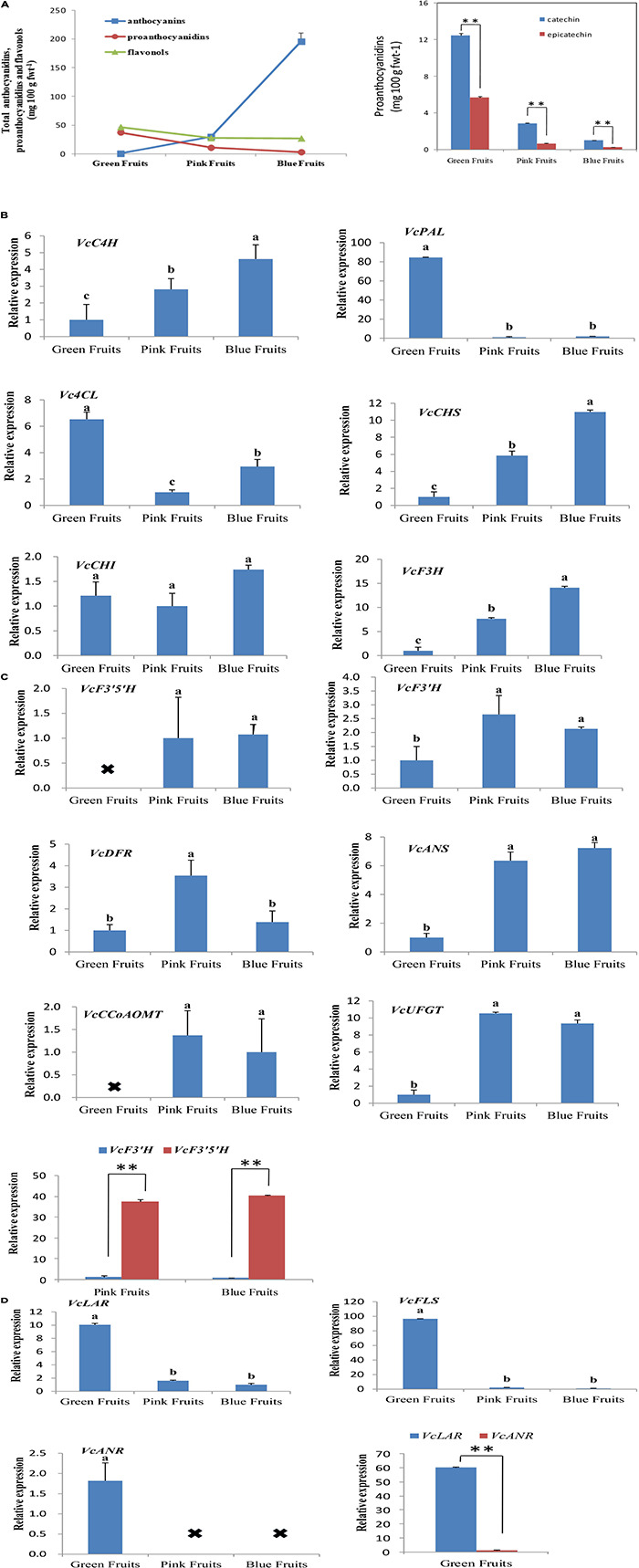
Analysis of the expression of genes associated with flavonoid synthesis and accumulated flavonoids. **(A)** Liquid chromatography was used for quantitation of flavonoids content. Liquid chromatography-mass spectrometry was used for qualitative testing of flavonoids. Proanthocyanidin content at different developmental stages in blueberries. fwt, fresh weight. Asterisks denote *t*-test significance: ^∗∗^*P* < 0.01. The mean ± SE of biological triplicates were taken for every value. **(B–D)** All values were calculated based on the geometric mean of the housekeeping genes, *VcGADPH* and *VcSAND*, at each stage. Asterisks denote *t*-test significance: ^∗∗^*P* < 0.01. Different English letters represents that the one-way ANOVA difference was significant (*P* < 0.05). The mean ± SE of biological triplicates were taken for every value.

To study the correlation between expression patterns of flavonoid synthesis genes and accumulated flavonoids in fruits, this study employed HPLC-DAD-ESI-MS to quantitate anthocyanin, flavonol, and proanthocyanidin content in fruits from three developmental stages. Total anthocyanin content showed an increasing trend by development stage, while the total flavonol and proanthocyanidin content decreased (Figure 2A).

The anthocyanin synthesis pathway is an important branch of the flavonoid synthesis pathway, which is relatively conserved in higher plants, and its process involves multiple complex enzymatic reactions (Supplementary Figure 2). Most studies on flavonoid synthesis have been in model plants such as apples and peaches, whereas fewer studies have used blueberries (Zhou et al., 2015; An et al., 2017a, An et al., 2020b). To validate the bioinformatics analysis results of annotated genes in the sequencing libraries and study the expression of genes in the phenylalanine metabolic pathways in blueberries, we used homology alignment and phylogenetic analysis to analyze structural genes of 15 main enzymes involved in anthocyanin synthesis pathway: *PAL, C4H, 4CL, CHS, CHI* (chalcone isomerase), *F3H* (flavanone 3′-hydroxylase), *F3*′*H* (flavonoid 3′ -hydroxylase), *F3*′*5*′*H* (flavonoid 3′, 5′-hydroxylase), *DFR, ANS/LDOX* (leucoanthocyanidin dioxygenase), *UFGT* (UDP-glucose: flavonoid 3-O-glucosyltransferase), *CCoAOMT* (caffeoyl-CoA O-methyltransferase), *FLS* (flavonol synthase), *ANR* (anthocyanidin reductase) and *LAR* (leucoanthocyanidin reductase). These genes have the largest FPKM (Fragments Per Kilobase of transcript per Million mapped reads).

Fluorescence quantitative PCR was used to quantitate the expression status of the structural genes and regulatory genes in flavonoid synthesis during the three developmental stages of blueberries. In this study, we referenced the results from the literature (Zhou et al., 2015) and selected *VcGADPH* and *VcSAND* as internal reference genes. The geometric means of the Ct values of these two genes were used as calibration values to analyze the relative expression of other genes. Supplementary Table 2 shows the annotated functions and FPKM (fragments per kb per million) values obtained from transcriptome sequencing of these genes. Supplementary Table 3 shows the primer sequences for qPCR.

Fluorescence qPCR results showed that the genes for flavonoid synthesis exhibited varied trends in expression during the three developmental stages of fruits. We first analyzed the expression of VcC4H, *VcPAL*, *Vc4CL, VcCHS, VcCHI*, and *VcF3H* (Figure 2B). These genes are located upstream of the phenylalanine metabolic pathway and the enzymes encoded by them can catalyze the synthesis of anthocyanins, proanthocyanidins, and flavonols. The results of qPCR showed that with the exception of *VcPAL* and *Vc4CL*, the expression of the remaining four genes was highest in the most mature fruits (blue fruits). *VcPAL* had the highest expression in the least mature (green) fruits and extremely low expression in pink and blue fruits. The expression of *Vc4CL* was the highest in green fruits, and lowest in pink fruits (Figure 2B).

Next, we analyzed six genes that encode for anthocyanin biosynthesis (*VcF3*′*H, VcF3*′*5*′*H, VcDFR, VcANS, VcUFGT*, and *VcCCoAOMT*). In the middle of the anthocyanin synthetic pathway, *VcF3*′*5*′*H* and *VcF3*′*H* are branch points as *VcF3*′*5*′*H* can regulate the biosynthesis of delphinidin, petunidin, and malvidin in blueberries whereas *VcF3*′*H* can regulate the synthesis of cyanidin and peonidin. In all three fruit developmental stages, *VcF3*′*5*′*H* and *VcANS* had persistently elevated levels whereas *VcF3*′*H, VcDFR, VcUFGT*, and *VcCCoAOMT* varied; levels were low in immature (green) fruit and high in mature (blue) fruit, then even higher in intermediate (pink) fruits (Figure 2C). Interestingly, in pink and blue fruits, the expression of *VcF3*′*5*′*H* was 31- and 40-fold that of *VcF3*′*H* (Figure 2C). This was also verified in quantitation of anthocyanin contents. In blue fruits, the total content of delphinidin, petunidin, and malvidin was 8.1 times that of the total content of cyanidin and peonidin (Table 2). These results suggest that anthocyanin biosynthesis mainly occurs during the fruit maturation phase in “Duke” blueberries, particularly the synthesis of delphinidin, petunidin, and malvidin, and also express the *VcF3*′*5*′*H* gene was upregulated in colored fruits, which changed the ratio of different anthocyanins.

**TABLE 2:** Types and contents of anthocyanins in fruits at different developmental stages in blueberries.

	**Green fruits**	**Pink fruits**	**Blue fruits**
Delphinidin 3-galactoside	0.12 ± 0.00c	9.07 ± 0.03b	43.47 ± 0.06a
Delphinidin 3-glucoside	×	0.14 ± 0.00b	0.62 ± 0.03a
Delphinidin 3-arabinoside	0.05 ± 0.00c	4.83 ± 0.13b	21.34 ± 0.04a
Cyanidin 3-galactoside	0.16 ± 0.01c	2.70 ± 0.02b	10.74 ± 0.16a
Cyanidin 3-glucoside	×	×	0.23 ± 0.00a
Cyanidin 3-arabinoside	0.05 ± 0.01c	1.22 ± 0.04b	4.80 ± 0.11a
Petunidin 3-galactoside	0.04 ± 0.00c	4.23 ± 0.02b	28.34 ± 1.01a
Petunidin 3-glucoside	×	0.10 ± 0.00b	0.58 ± 0.03a
Petunidin 3-arabinoside	×	2.05 ± 0.09b	11.58 ± 0.50a
Peonidin 3-galactoside	×	0.50 ± 0.01b	4.04 ± 0.08a
Peonidin 3-glucoside	×	×	0.31 ± 0.04a
Peonidin 3-arabinoside	×	0.16 ± 0.00b	1.31 ± 0.08a
Malvidin 3-galactoside	×	3.00 ± 0.13b	47.34 ± 0.10a
Malvidin 3-glucoside	×	0.08 ± 0.00b	1.25 ± 0.08a
Malvidin 3-arabinoside	×	1.53 ± 0.05b	19.16 ± 0.85a

*Values shown are the mean ± SE from three biological replicates. ×Indicates the substance was not detected. Different English letters represents that the one-way ANOVA difference was significant (P < 0.05).*

Then, we analyzed the *VcFLS* gene that regulates flavonol synthesis. *VcFLS* had the highest expression in green fruits and extremely low expression in pink and blue fruits (Figure 2D). This result was consistent with quantitation of flavonol content, which showed that flavonol contents in green fruits were approximately 1.69 times greater than in pink or blue fruits (Figure 2A). Subsequently, we analyzed two genes that regulate proanthocyanidin biosynthesis, *VcANR* and *VcLAR*, which were mainly expressed in green fruits and the expression of *VcLAR* was approximately 60 times that of *VcANR* (Figure 2D). The quantitation results of proanthocyanidins in fruits showed that catechin content was 2.19 times that of epicatechin content in green fruits (Figure 2A). The results suggest that the differential expression of *VcLAR* and *VcANR* may be responsible for differences in catechin and epicatechin contents. These results suggest that the biosynthetic characteristics of flavonols and proanthocyanidins are relatively similar as they are mainly carried out in immature fruits and there is almost little or no biosynthesis in mature fruits. Of these enzymes, *VcLAR* may play a dominant role in proanthocyanidin synthesis in green fruits compared with *VcANR*.

**FIGURE 3:**
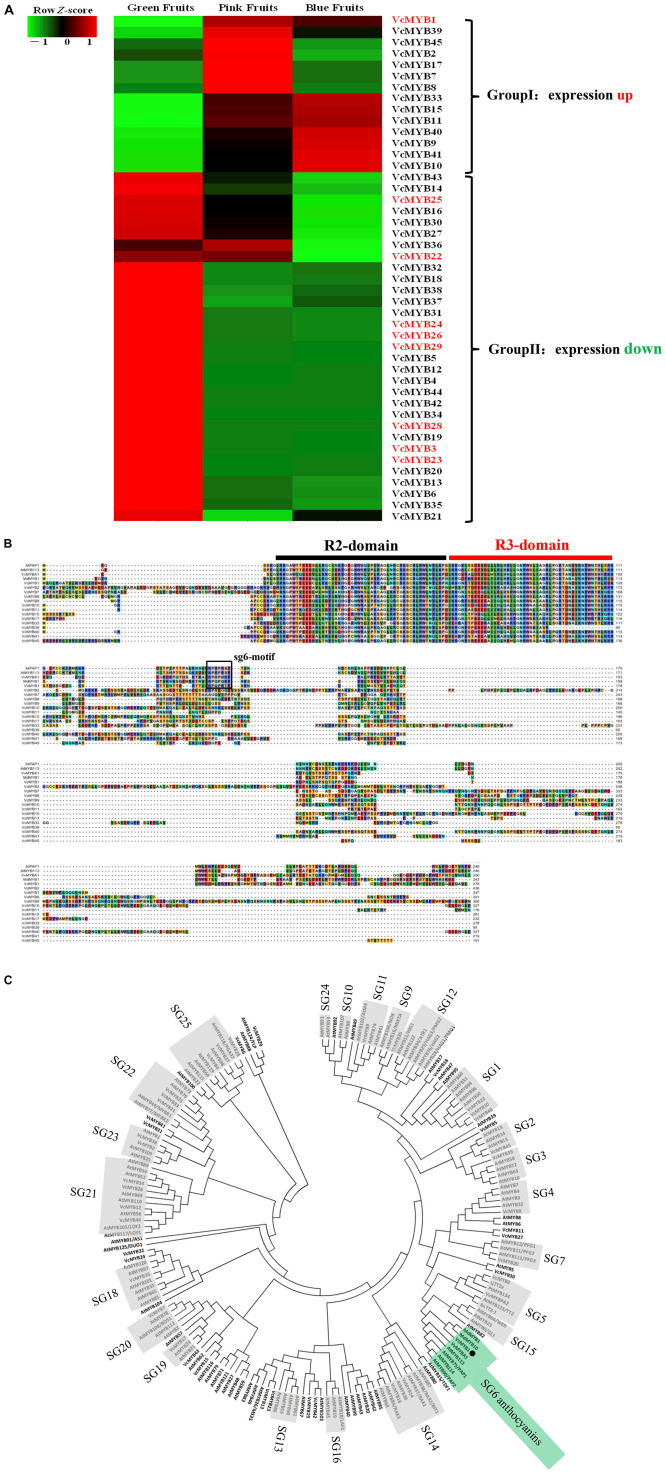
Sequence alignment and phylogenetic analysis of VcMYB. **(A)** Heat map depicting the various MYBs, qRT-PCR analysis was also carried out for the genes in red. **(B)** Protein alignment of Vc MYB and its homologs in *Arabidopsis, Vitis vinifera* and *Malus domestica*, The sg6-motif in boxed. **(C)** Phylogenetic analysis of VcMYB and 138 other plants MYB protein sequences obtained from the NCBI database.VcMYB1 is denoted by the black spot. MYB of *Arabidopsis* (AtMYB0: At3g27920; AtMYB1: At3g09230; AtMYB10: At3g12820; AtMYB100: At2g25230; AtMYB101: At2g32460; AtMYB102: At4g21440; AtMYB103: At1g63910; AtMYB104: At2g26950; AtMYB105: At1g69560; AtMYB106: At3g01140; AtMYB107: At3g02940; AtMYB108: At3g06490; AtMYB109: At3g55730; AtMYB11: At3g62610;AtMYB110: At3g29020 AtMYB111: At5g49330; AtMYB112: At1g48000; AtMYB113: At1g66370; AtMYB114: At1g66380; AtMYB115: At5g40360; AtMYB116: At1g25340; AtMYB117: At1g26780; AtMYB118: At3g27780; AtMYB119: At5g58850; AtMYB12: At2g47460; AtMYB120: At5g55020; AtMYB121: At3g30210; AtMYB122: At1g74080; AtMYB123: At5g35550; AtMYB124: At1g14350; AtMYB125: At3g60460; AtMYB13: At1g06180; AtMYB14: At2g31180; AtMYB15: At3g23250; AtMYB16: At5g15310; AtMYB17:At3g61250; AtMYB18: At4g25560; AtMYB19: At5g52260; AtMYB2: At2g47190; AtMYB20: At1g66230; AtMYB21: At3g27810; AtMYB22: At5g40430; AtMYB23: At5g40330; AtMYB24: At5g40350; AtMYB25: At2g39880; AtMYB26: At3g13890; AtMYB27: At3g53200; AtMYB28: At5g61420; AtMYB29: At5g07690; AtMYB3: At1g22640; AtMYB30: At3g28910; AtMYB31: At1g74650; AtMYB32: At4g34990; AtMYB33: At5g06100; AtMYB34: At5g60890; AtMYB35: At3g28470; AtMYB36: At5g57620; AtMYB37: At5g23000; AtMYB38: At2g36890; AtMYB39: At4g17780; AtMYB4: At4g38620; AtMYB40: At5g14340; AtMYB41: At4g28110; AtMYB42: At4g12350; AtMYB43: At5g16600; AtMYB44: At5g67300; AtMYB45: At3g48920; AtMYB46: At5g12870; AtMYB47: At1g18710; AtMYB48: At3g46130; AtMYB49: At5g54230; AtMYB5: At3g13540; AtMYB50: At1g57560; AtMYB51: At1g18570; AtMYB52: At1g17950; AtMYB53: At5g65230; AtMYB54: At1g73410; AtMYB55: At4g01680; AtMYB56: At5g17800; AtMYB57: At3g01530; AtMYB58: At1g16490; AtMYB59: At5g59780; AtMYB6: At4g09460; AtMYB60: At1g08810; AtMYB61: At1g09540; AtMYB62: At1g68320; AtMYB63: At1g79180; AtMYB64: At5g11050; AtMYB65: At3g11440; AtMYB66: At5g14750; AtMYB67: At3g12720; AtMYB68: At5g65790; AtMYB69: At4g33450; AtMYB7: At2g16720; AtMYB70: At2g23290; AtMYB71: At3g24310; AtMYB72: At1g56160; AtMYB73: At4g37260; AtMYB74: At4g05100; AtMYB75: At1g56650; AtMYB76: At5g07700; AtMYB77: At3g50060; AtMYB78: At5g49620; AtMYB79: At4g13480; AtMYB8: At1g35515; AtMYB80: At5g56110; AtMYB81: At2g26960; AtMYB82: At5g52600; AtMYB83: At3g08500; AtMYB84: At3g49690; AtMYB85: At4g22680; AtMYB86: At5g26660; AtMYB87: At4g37780; AtMYB88: At2g02820; AtMYB89: At5g39700; AtMYB9: At5g16770; AtMYB90: At1g66390; AtMYB91: At2g37630; AtMYB92: At5g10280; AtMYB93: At1g34670; AtMYB94: At3g47600; AtMYB95: At1g74430;AtMYB96: At5g62470; AtMYB97: At4g26930; AtMYB98: At4g18770; AtMYB99: At5g62320; MdMYB10: *M. domestica*, ABB84753; LjTT2a: *Lotus japonicas*, BAG12893; PtMYB134: *P. tremuloides*, FJ573151; VvMYBPA2: *Vitis vinifera*, EU919682; BnTT2-1: *Brassica napus*, DQ778643; MdMYB1: Malus domestica, DQ886414.

In the blueberry transcriptome data, we obtained a total of 45 blueberry MYB gene sequences named VcMYB1-45, respectively. A heat map analysis of these gene sequences revealed that 14 MYB genes were up-regulated in the stages of green fruit to pink fruit or from green fruit to blue fruit, and 31 MYB genes were down-regulated at the above stage (Figure 3A). The amino acid sequences of R2R3 MYB transcription factors in other plants that regulate anthocyanin synthesis, such as grapes (*VvMYBA1*), apples (*MdMYB10*), and *Arabidopsis (PAP1*) were used as probes for alignment and screening of the MYB amino acid sequence of up-regulated gene expression in blueberries. It was found that only *VcMYB1* contained the sg6-motif among the genes up-regulated in blueberries (Figure 3B). Studies have shown that the sg6 motif can promote the synthesis of plant anthocyanins (Stracke et al., 2001; Muñoz et al., 2021). For further verification, Phylogenetic analysis of all the excavated VcMYB protein sequences and homologous sequences of other 138 species of plants showed that only the *VcMYB1* gene has extremely high homology with the *Arabidopsis* SG-6 R2R3 MYB protein (Figure 3C).

To further verify the reliability of the blueberry transcriptome data. Fluorescence qPCR results using 9 blueberry VcMYB transcription factors with larger FPKM showed that the expression trends of *VcMYB1* were similar to the expression trends of the six genes that regulate anthocyanin synthesis, such as *VcDFR* and *VcF3*′*5*′ *H*, which are mainly expressed in colored fruits. *VcMYB23, VcMYB26*, and *VcMYB28* are almost only expressed in green fruits. *VcMYB25, VcMYB27*, and *VcMYB29* exhibited a continuously declining trend. *VcMYB24* levels varied; in green fruit *VcMYB24* was high; in mature fruits it was lower. *VcMYB22* expression varied; it was highest in immature (green) fruits, lower in pink fruits, and higher in the mature (blue) fruits (Supplementary Figure 3). The results suggest that the upregulated expression of *VcMYB1* in pink and blue fruits may promote the synthesis of anthocyanins.

### Functional Validation of *VcMYB1*

**FIGURE 4:**
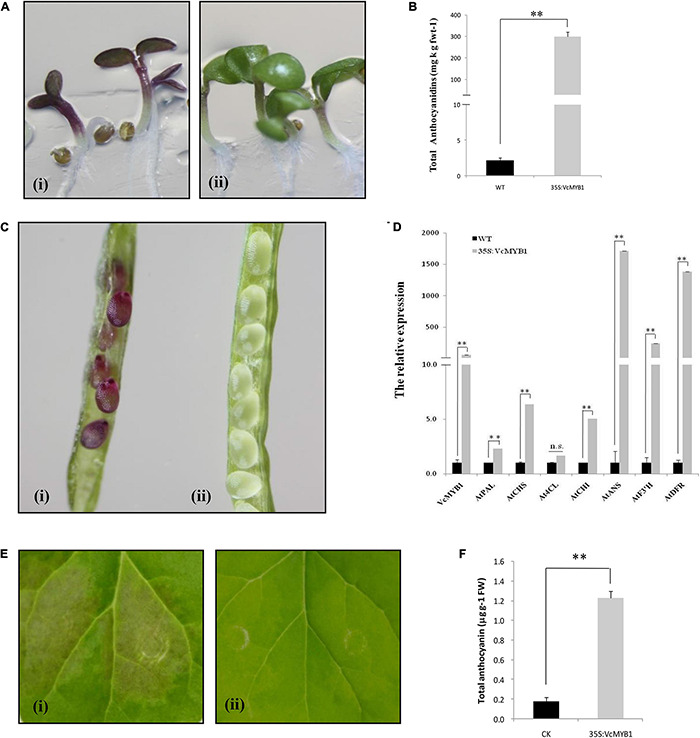
Functional analysis of *VcMYB1* in a heterologous system. **(A)** *Arabidopsis thaliana* (ecotype Columbia) plants overexpressing (i) 35S:VcMYB1 and (ii) wild-type (WT) *A. thaliana*. **(B)** Anthocyanin content of *A. thaliana* plants overexpressing 35S:VcMYB1 and wild-type *A. thaliana*. Asterisks denote *t*-test significance: ^∗∗^*P* < 0.01. The mean ± SE of biological triplicates were taken for every value. **(C)** Seeds of *A. thaliana* plants overexpressing (i) 35S:VcMYB1 and (ii) WT *A. thaliana*. **(D)** Relative expression of *VcMYB1, AtPAL, AtCHS, At4CL, AtCHI, AtANS, AtF3*′*H*, and *AtDFR* in *A. thaliana* plants overexpressing 35S:VcMYB1 and wild-type *A. thaliana*. All values were calculated based on the housekeeping gene *AtUBQ*. Asterisks denote *t*-test significance: ^∗∗^*P* < 0.01. n.s means no significant differences. The mean ± SE of biological triplicates were taken for every value. **(A,C)** Are one of the four T2 transgenic stains and all strains exhibit the phenotype shown by **(A,C)**. **(E)** Tobacco (*Nicotiana benthamiana*) leaves on day 7 after injection. (i) 35S:VcMYB1, (ii) Wild-type. **(F)** Anthocyanin content in tobacco leaves on Day 7 after injection. The mean ± SE of biological triplicates were taken for every value. Asterisks denote *t*-test significance: ^∗∗^*P* < 0.01.

Given that the expression patterns of *VcMYB1* and anthocyanin-synthesis genes are similar, i.e., relatively high expression in colored fruits and *VcMYB1* has relatively high homology and similarity to MYB transcription factors in other plants that regulate anthocyanin synthesis (Figures 3B,C), we hypothesized that *VcMYB1* may positively regulate anthocyanin synthesis. To investigate whether *VcMYB1* can promote anthocyanin synthesis, we used a heterologous expression system and validated the function of *VcMYB1* in *Arabidopsis* (ecotype Columbia) under the control of CaMV-35S. Results showed that under the same conditions, red pigmentation was present in *Arabidopsis* seeds (8–10 days after flowering), cotyledons, and hypocotyls overexpressed *35S_*pro*_:VcMYB1* whereas red pigmentation was absent in wild-type (WT) *Arabidopsis* in these three tissues (Figures 4A,C). Anthocyanin contents in transgenic plants and control plants were 311.61 mg/kg fresh weight and 2.82 mg/kg fresh weight, respectively. Thus, anthocyanin contents in plants overexpressing *VcMYB1* was approximately 110 times the expression levels in control plants (Figure 4B). To further understand the molecular mechanisms by which *VcMYB1* regulates anthocyanin synthesis in *Arabidopsis*, we extracted RNA from *35S_*pro*_:VcMYB1 Arabidopsis* and WT *Arabidopsis*. Fluorescence qPCR was used to measure the expression of structural genes for anthocyanin synthesis in *Arabidopsis* such as *AtPAL, AtCHS* and *AtDFR*, and *VcMYB1*. Except for *At4CL*, expression of all other genes in the transgenic plants was significantly higher than in WT plants (Figure 4D). This suggests that *VcMYB1* affects anthocyanin synthesis in *Arabidopsis* by regulating structural genes that are related to anthocyanin synthesis.

To confirm the function of *VcMYB1*, we conducted transient expression experiments and validated the function of *VcMYB1* in tobacco (*Nicotiana benthamiana*). *35S_*pro*_:VcMYB1* was transformed into Agrobacterium before injecting into tobacco leaves. Agrobacterium that were transformed with empty vectors were used as controls. Results showed that 4 days after injection, tobacco leaves overexpressing *VcMYB1* were colored whereas no color changes were observed in the control (Figure 4E). Anthocyanin content measurement results showed that the total anthocyanin content in tobacco leaves overexpressing *VcMYB1* was 6.8-fold higher than in the control leaves (Figure 4F). The above data shows that *VcMYB1* can positively regulate anthocyanin synthesis.

**FIGURE 5:**
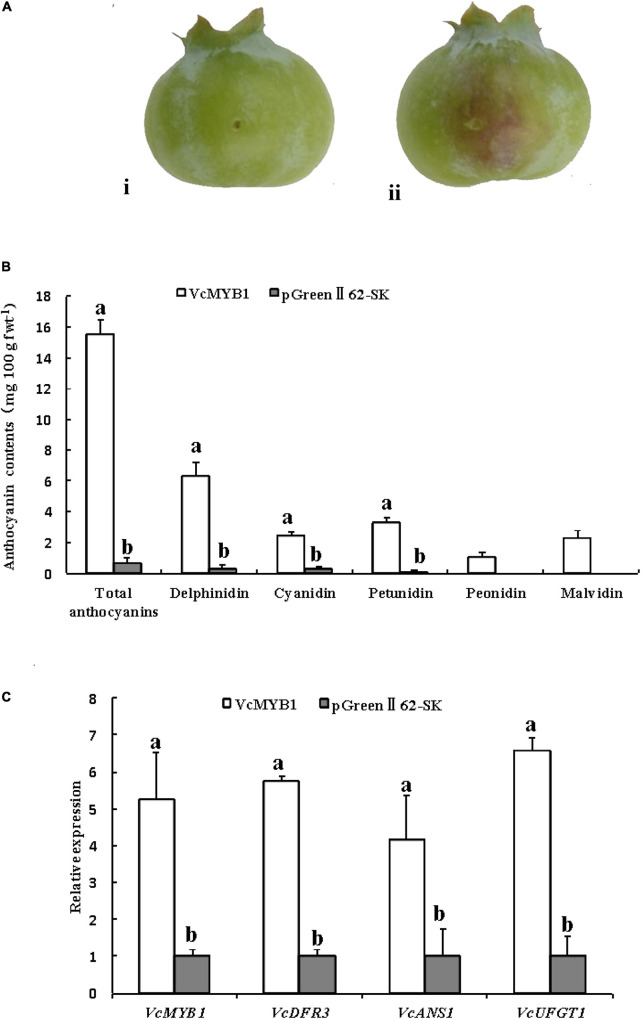
Functional analysis of *VcMYB1* in blueberry. **(A)** blueberry overexpressing (i) pGreenII62-SK fruit and (ii) VcMYB1-pGreenII62-SK (VcMYB1) fruit. **(B)** Anthocyanin content of fruits overexpressing VcMYB1-pGreenII62-SK (VcMYB1) and pGreenII62-SK fruit. The mean ± SE of biological triplicates were taken for every value. Different English letters represents that the *t*-test difference was significant. *P* < 0.05. **(C)** Relative expression of *VcMYB1, VcDFR3, VcANS1*, and *VcUFGT1* in fruits overexpressing VcMYB1-pGreenII62-SK (VcMYB1) and pGreenII62-SK fruit. The mean ± SE of biological triplicates were taken for every value. Different English letters represents that the *t*-test difference was significant *P* < 0.05.

Moreover, vector-mediated overexpression was conducted using 45 days blueberries. VcMYB1-pGreenII62-SK vectors (VcMYB1) were generated, and pGreenII62-SK were used as a control. Compared with the empty vector control (pGreenII62-SK), overexpression of *VcMYB1* promoted anthocyanin accumulation in the blueberry shin around the injection sites (Figures 5A,B), The total content of delphinidin, petunidin, and malvidin was higher than the total content of cyanidin and peonidin, consistent with Supplementary Table 2. The expression levels of *VcMYB1, VcDFR3, VcANS1* and *VcUFGT1* were elevated in the VcMYB1-pGreenII62-SK vectors injection areas compared with the controls (Figure 5C). Therefore, these results demonstrated that *VcMYB1* played a positive role in anthocyanin synthesis and fruit coloration.

### *VcMYB1* Activates *VcDFR* Promoters

**FIGURE 6:**
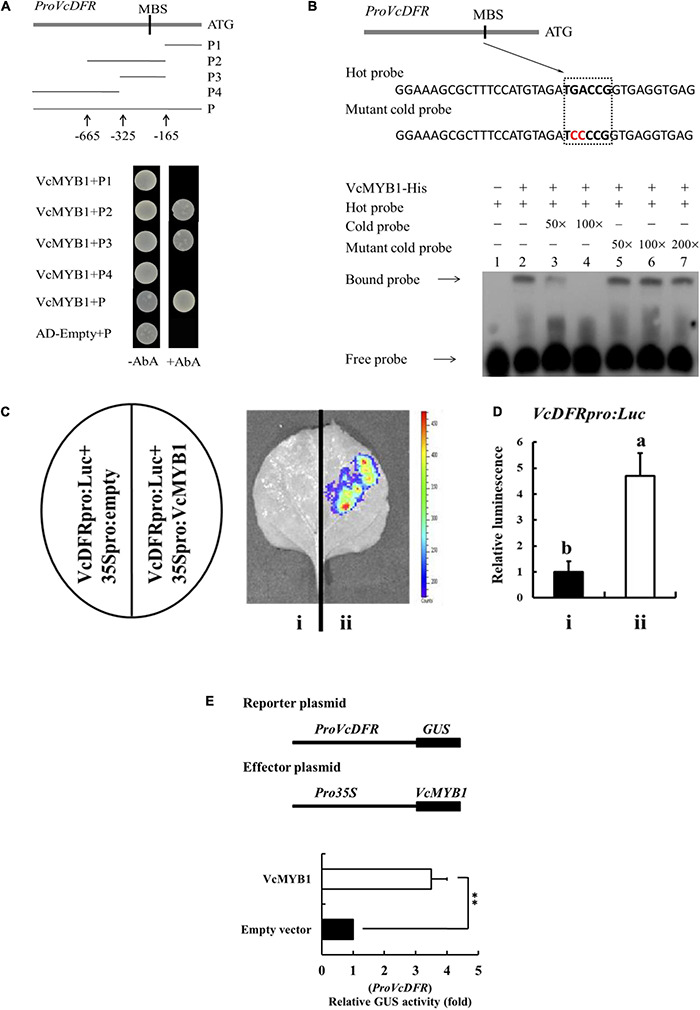
VcMYB1 can directly bind to and activate the *VcDFR* promoter. **(A)** Y1H analysis showed that VcMYB1 can bind to *VcDFR* promoter fragments that contain the MBS motif. The *VcDFR* promoter was divided into four segments (P1–P4). AbA (Aureobasidin A) is a growth inhibitor of yeast cells and was used as a screening marker. The screening concentration for AbA was 100 ng/mL. The empty vector and the full-length *VcDFR* promoter were used as a negative control. **(B)** EMSA analysis showed that VcMYB1 can bind to the MBS motif on the *VcDFR* promoter. The hot probe contains a *VcDFR* promoter with a biotinylated MBS motif and the unbiotinylated cold probe was used as a competitive probe (concentration of 50- and 100-fold to the hot probe). The mutant cold probe is an unbiotinylated hot probe in which three nucleotides were mutated. **(C)** Firefly luciferase complementation assay analysis showed that VcMYB1 can activate *VcDFR* promoter activity in tobacco (N. benthamiana) leaves. (i) *VcDFR_*pro*_:Luc* + 35Spro:empty, (ii) *VcDFR_*pro*_:Luc* + 35S_*pro*_:VcMYB1. **(D)** Quantitative analysis of luminescence intensity in C. The value for a *VcDFR_*pro*_:Luc* was set to 1. The mean ± SE of biological triplicates were taken for every value. Different English letters represents that the *t*-test difference was significant. *P* < 0.01 **(E)** GUS activity analysis showed that VcMYB1 can activate *VcDFR* promoter activity in tobacco (N. benthamiana) leaves. VcMYB1 effector vector and a reporter vector containing the *VcDFR* promoter were co-injected into wild-type tobacco leaves to analysis their effects on GUS activity. Asterisks denote *t*-test significance: ^∗∗^*P* < 0.01. The mean ± SE of biological triplicates were taken for every value.

Because *VcMYB1* can promote anthocyanin synthesis, we predicted that *VcMYB1* may directly regulate the expression of structural genes in anthocyanin synthesis to regulate anthocyanin synthesis. Previous studies have reported that MYB function by binding to the promoters of their target genes to modulate their expressions (Takos et al., 2006; Ban et al., 2007; Espley et al., 2007; An et al., 2020a). We employed chromosome walking techniques to clone the promoter sequence of *VcDFR, VcF3*′*5*′ *H* and *VcUFGT*, which was found to have a length of 834, 1,828, and 2,362 bp respectively. Unfortunately, the promoter sequence of *VcANS* is not available. This promoter was inserted into a pAbAi vector before transformation into a Y1HGoldstrain. The open reading frame of *VcMYB1* was inserted into a pGADT7 vector. The Y1H assay was used to analyze whether VcMYB1 can interact with these promoters. Results showed that VcMYB1 can bind the MYB binding site (MBS) motifs in the *VcDFR* promoter (Figure 6A). For further research, we used the promoter prediction website^10^ to predict and analyze the *VcDFR*, *VcF3*′*5*′*H* and *VcUFGT* promoter sequences. It was found that only the *VcDFR* promoter sequence contained the MBS motif. In the anthocyanin synthesis pathway, the action site of *VcDRF* is adjacent to the action sites of the other three structural genes. Therefore, it is speculated that the expression of *VcDRF* will promote their expression. We also used a prokaryotic expression system to induce and purify the VcMYB1 protein and used electrophoretic mobility shift assay (EMSA) to validate the results of the Y1H assay. Results showed that VcMYB1 can bind to the biotinylated MBS motif on the *VcDFR* promoter (Figure 6B).

To investigate whether *VcMYB1* can activate the *VcDFR* promoter, we carried out firefly luciferase (Luc) complementation experiments for validation. We inserted the *VcDFR* promoter upstream of the Luc gene to generate *VcDFR_*pro*_:Luc*. *VcMYB1* was inserted downstream of the 35S promoter to generate *35S_*pro*_:VcMYB1*. Both vectors were transformed into Agrobacterium before co-injection into tobacco leaves. Results showed that luminescence signals could be detected in tobacco plants that were co-injected with *VcDFR_*pro*_:Luc* and *35S_*pro*_:VcMYB1* whereas no luminescence signals were detected in the control (*VcDFR_*pro*_:Luc* + 35S_*pro*_:empty) (Figures 6C,D). Following that, we validated the regulation of the *VcDFR* promoter by *VcMYB1* in tobacco leaves by analyzing the activity of the β-glucuronidase (GUS) reporter gene. The results showed that co-transforming *VcDFR_*pro*_:Luc* and *35S_*pro*_:VcMYB1* into tobacco leaves elevated *VcDFR* promoter activity (Figure 6E). Therefore, we believe that VcMYB1 directly binds to the promoter of *VcDFR* to activate the expression of this gene, thereby promoting anthocyanin synthesis.

## Discussion

Blueberries contain large amounts of diverse flavonoids, which reduce reactive oxygen species generation and they enhance the ability of plants to resist biological and non-biological stressors (Cascaes Márcia et al., 2021; Mu et al., 2021). But there were very few studies on the molecular mechanisms of their synthesis or their metabolism. This study is the first to employ SMRT combined with Illumina sequencing technology to carry out transcriptome sequencing of three fruit developmental stages in blueberries to improve the abundance of transcripts as much as possible in order to understand the mechanisms for flavonoid synthesis in fruits.

This study is the first to employ SMRT combined with Illumina sequencing technology to carry out transcriptome sequencing of three fruit developmental stages in blueberries. Results showed that compared with the assembled data obtained from Illumina sequencing, better data quality and longer transcripts were obtained from SMRT sequencing. This was also found to be the case in studies by Xu et al. (2015) and Li et al. (2017), i.e., The amount of data acquired by Illumina sequencing was approximately 2.4 times that acquired by SMRT sequencing. This was not consistent with the results by Li et al. (2017) who found a greater number of annotated genes by Illumina sequencing compared with SMRT sequencing. This inconsistency may be due to species specificity. Therefore, combining these two types of sequencing techniques can be used to obtain abundant and more intact transcriptome information of blueberries during fruit development. Our study obtained a large number of structural genes that participate in flavonoid synthesis through combination of Illumina and SMRT sequencing methods. The number of genes involved in flavonoid synthesis that were obtained by Sun et al. (2015); Li Y. Y. et al. (2012), and Li et al. (2016) using Illumina sequencing technologies were lower than that obtained in this study (Supplementary Table 4). The results showed that compared with single assembly using the established Illumina sequencing method, SMRT sequencing greatly improved the assembly results of Illumina fragmentation. Compared with using only one sequencing technology (Illumina or SMRT), the integrated use of these two sequencing technologies can significantly enrich transcriptome information and increase the integrity of transcriptome data. The combination of these two sequencing technologies can thus enable more effective and greater acquisition of target transcriptome information. Specifically, we obtained full-length sequences of genes that are involved in flavonoid synthesis, showing this technique can aid the discovery and analysis of functional genes.

The results of the study showed that anthocyanin contents continuously increased, across the three fruit development stages (Figure 2A). In addition, this study also screened out a MYB transcription factor that regulates anthocyanin synthesis, VcMYB1. From this, we can see that “Duke” blueberries mainly synthesize anthocyanins in colored fruits (pink and blue fruits). The anthocyanin synthesis genes (*VcF3*′*H, VcF3*′*5*′*H, VcDFR, VcANS, VcUFGT* and *VcCCoAOMT*) were all mainly expressed in colored fruits (Figure 2C). This result was consistent with the results of other studies, i.e., the upregulated expression of these six genes can promote plant coloring and increase anthocyanin content (Bogs et al., 2006; Yao et al., 2017; Ma et al., 2021). The differential expression of *F3*′*5*′*H* and *F3*′*H* genes can affect the type of anthocyanins synthesized in plants and different colors are presented (Holton et al., 1993a; Han et al., 2010). Our study found that *VcF3*′*5*′*H* and *VcF3*′*H* are mainly expressed in colored fruits in “Duke” blueberries, but expression is vastly different (Figure 2C). This explains why the total content of delphinidin, petunidin, and malvidin in pink and blue fruits was significantly higher than the total content of cyanidin and peonidin (Table 2). This result is supported by previous studies of flavonoid content in blueberries (Lätti et al., 2010; Zifkin et al., 2012).

The WBM (WD40, BHLH, and MYB) complex regulates flavonoid synthesis through interactions between its components as well as binding to the promoters of structural genes for anthocyanin, flavonol, and proanthocyanidin synthesis, such as *DFR* and *UFGT* (Grotewold et al., 2000; Mehrtens et al., 2005; Stracke et al., 2007; Xie et al., 2012; Zifkin et al., 2012). Among the R2R3 MYB transcription factors, AtMYBs (AtMYB75, AtMYB90, AtMYB113, and AtMYB144), MdMYB1 and MdMYB10 can promote the expression of genes encoding key anthocyanin biosynthetic enzymes, such as *DFR* and *ANS* (Dooner et al., 1991; Takos et al., 2006; Ban et al., 2007; Espley et al., 2007; Antonio et al., 2008; Li et al., 2014; An et al., 2018; An et al., 2020a). Our study screened out one R2R3 MYB transcription factor, *VcMYB1* from the transcriptome library. VcMYB1 and these MYB transcription factors have a high degree of homology (Figure 3A). Fluorescence quantitative PCR analysis results showed that the expression pattern of *VcMYB1* is similar to the expression patterns of multiple genes that regulate anthocyanin synthesis, which are mainly highly expressed in colored fruits. Functional validation experiments also proved that *VcMYB1* can promote anthocyanin synthesis in *Arabidopsis*, tobacco plants and green blueberry fruits (Figures 4, 5). In addition, Y1H assay, EMSA, luciferase complementation, and GUS activity experiments showed that VcMYB1 can bind to the promoter of *VcDFR*, which activates the promoter. This is similar to the results from many studies (Ban et al., 2007; James et al., 2017). VcMYB1 can promote the synthesis of anthocyanins by binding to the 5′-TGACCG-3′ sequence of the MBS motif of the *DFR* promoter. In *Arabidopsis*, the general MYB/bHLH protein complex binds to the *DFR* promoter sequence to promote anthocyanin synthesis (Ilona et al., 2004). In rice, MYB can bind to the promoter sequence of *DFR*, the sequences are CC(T/A)ACC (CCAACC) or AC(C/A)C(T/A)A(C/A)C (ACCTACC) (Kim et al., 2017). In apples, MdMYB1 can also bind to the MBS motif of the *DFR* promoter, and its binding sequence is 5′-GCCAGG-3′ which is different from VcMYB1 (An et al., 2018, 2020a). We speculate that this is related to the specificity of the species. Blueberries are non-climacteric plants, apples are climacteric plants, and rice and *Arabidopsis* do not have high-quality fleshy fruits. Therefore, we believe that in “Duke” blueberries, VcMYB1 can bind and activate the promoters of structural genes involved in anthocyanin synthesis, thereby positively regulating the biosynthesis of anthocyanins.

This study also carried out studies on genes involved in flavonol biosynthesis in blueberries. We found that *VcFLS* can regulate the biosynthesis of quercetin, myricetin and other flavonols. In this study, *VcFLS* is mainly expressed in green fruits and the expression of *VcFLS* in colored fruits is extremely low (Figure 2D). This causes flavonols to be mainly synthesized in green fruits (Figure 2A). This result was similar to the results by Holton et al. (1993b); Stracke et al. (2009), and Zifkin et al. (2012). VcLAR and VcANR can regulate proanthocyanidin synthesis (Tanner et al., 2003; Pang et al., 2013). In this study, the *VcLAR* and *VcANR* genes that regulate proanthocyanidin synthesis were only expressed in green fruits, with the expression of *VcLAR* being 60 times that of *VcANR* (Figure 2D). In addition, the contents of catechin were significantly higher than epicatechin. This shows that the high expression of *VcLAR* in green fruits resulted in accumulated of higher contents of proanthocyanidins. This result was similar to the results by Wang et al. (2017).

In summary, our study shows that flavonoid biosynthetic pathways are regulated in developing blueberry fruits, and that this regulation occurs at the level of gene expression. Our combinatorial approach to transcriptome analysis enabled us to generate full-length transcripts as well as providing evidence for flavonoid biosynthesis. Our work provides a new method for investigating the full-length transcriptome and secondary metabolism in other plants.

## Data Availability Statement

The original contributions presented in the study are publicly available. These data can be found here: National Center for Biotechnology Information (NCBI) BioProject database under BioProject ID: PRJNA757138.

## Author Contributions

YS conceived and designed the experiments. QT and YS performed the research and wrote the manuscript. QT, YS, F-MC, H-DL, and H-JZ analyzed the data. All authors contributed to the article and approved the submitted version.

## Conflict of Interest

The authors declare that the research was conducted in the absence of any commercial or financial relationships that could be construed as a potential conflict of interest.

## Publisher’s Note

All claims expressed in this article are solely those of the authors and do not necessarily represent those of their affiliated organizations, or those of the publisher, the editors and the reviewers. Any product that may be evaluated in this article, or claim that may be made by its manufacturer, is not guaranteed or endorsed by the publisher.

## Footnotes

1
http://www.agilent.com

2
https://www.thermofisher.com

3
http://www.sagescience.com

4
http://www.pacificbiosciences.com/devnet/

5
http://www.ncbi.nlm.nih.gov/BLAST/

6
http://smart.emblheidelberg.de/

7
http://www.takara.com.cn

8
http://www.clontech.com

9
http://www.bio-rad.com

10
http://bioinformatics.psb.ugent.be/webtools/plantcare/html/

## Supplementary Material

Supplementary Figure 1 Venn diagram of long non-coding RNA (lncRNA) sequences predicted by three different methods.

Supplementary Figure 2 Schematic diagram of flavonoid synthesis in blueberries, including anthocyanin, proanthocyanidin, and flavonol synthesis.

Supplementary Figure 3 Transcript abundance of MYB genes at different developmental stages in blueberries.

Supplementary Table 1 Statistics of sequences length from Illumina and SMRT sequencing data.

Supplementary Table 2 FPKM values and annotation of flavonoid-synthesis genes in blueberries.

Supplementary Table 3 List of primers used in this study.

Supplementary Table 4 Comparison of flavonoid synthesis-related genes in blueberries.
