# Supplementary material for: Single-Molecule Real-Time and Illumina Sequencing to Analyze Transcriptional Regulation of Flavonoid Synthesis in Blueberry
Source: Front Plant Sci. 2021 Sep 30;12:754325. doi: 10.3389/fpls.2021.754325 (PMC8514788; doi:10.3389/fpls.2021.754325)
Supplement: Supplementary Figure 1 — Venn diagram of long non-coding RNA (lncRNA) sequences predicted by three different methods. [file Data_Sheet_1.pdf]

Supplementary Material

1 Supplementary Figures and Tables

1.1 Supplementary Figures

Supplementary Figure 1

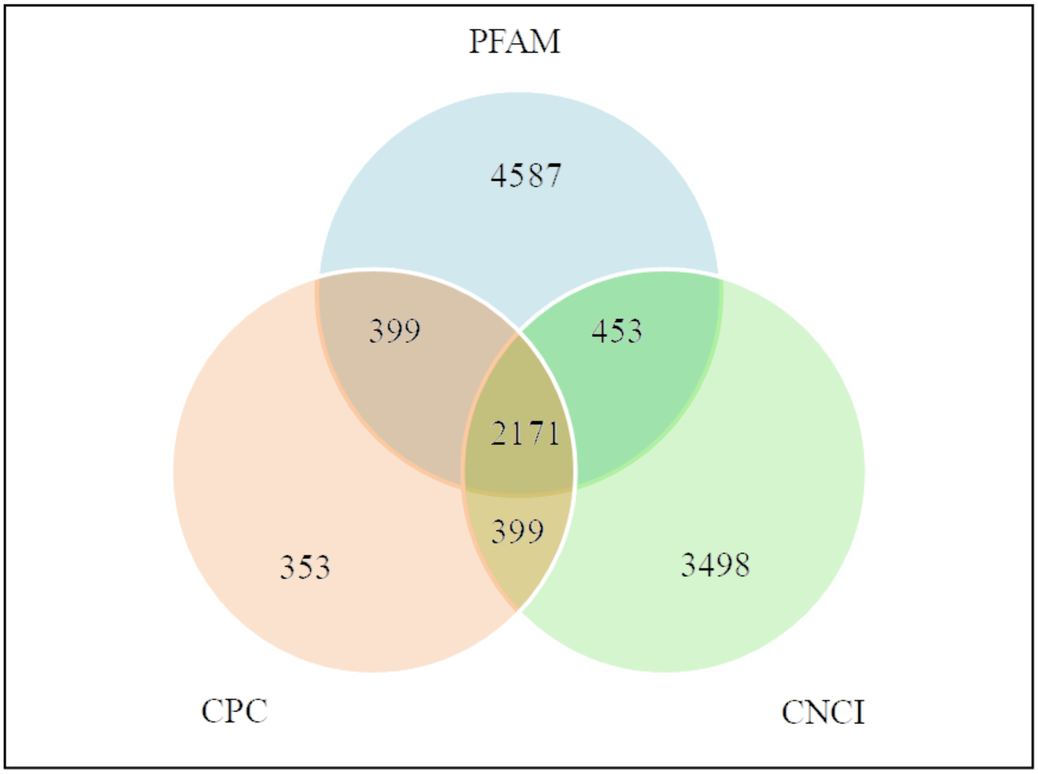

Supplementary Figure 1. Venn diagram of long non-coding RNA (lncRNA) sequences predicted by three different methods. Three methods (coding-non-coding index, CNCI; coding potential calculator, CPC; and Pfamscan, PFAM) were used for lncRNA prediction.

Supplementary Figure 2

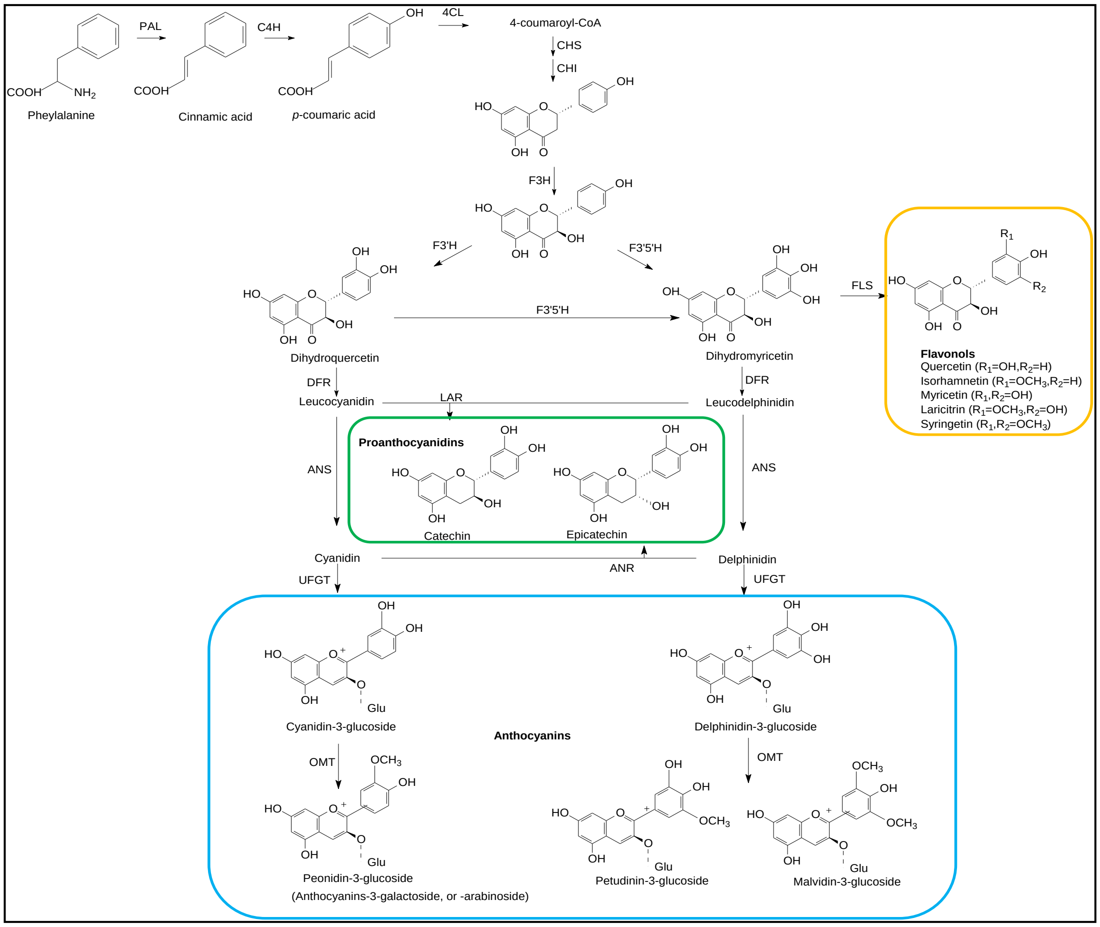

Supplementary Figure 2. Schematic diagram of flavonoid synthesis in blueberries, including anthocyanin, proanthocyanidin, and flavonol synthesis. Enzymes: PAL, phenylalanine ammonia lyase; C4H, cinnamate 4-hydroxylase; 4CL, 4-coumarate--CoA ligase; CHS, chalcone synthase; CHI, chalcone isomerase; F3H, flavanone 3-hydroxylase; F3′ H, flavonoid 3′ -hydroxylase; F3′ 5′ H, flavonoid 3′ ,5′ -hydroxylase; DFR, dihydroflavonol-4-Reductase; ANS/LDOX, leucoanthocyanidin dioxygenase; UFGT, UDP-glucose: flavonoid 3-O-glucosyltransferase; CCoAOMT, caffeoyl-CoA O-methyltransferase; FLS, flavonol synthase; ANR, anthocyanidin reductase; LAR, leucoanthocyanidin reductase.

Supplementary Figure 3

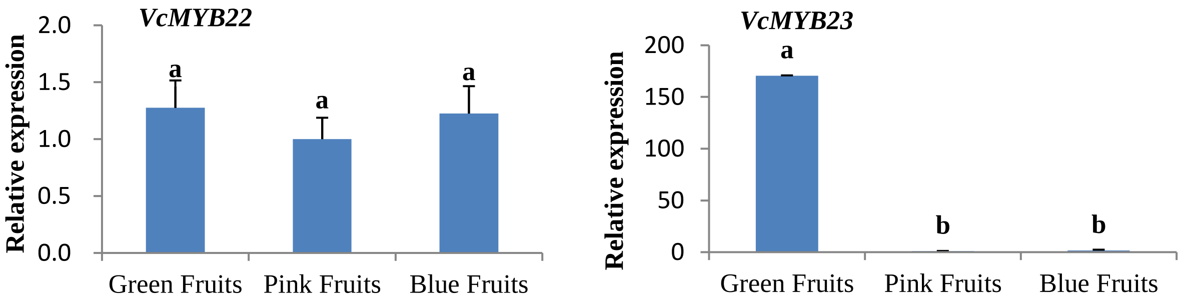

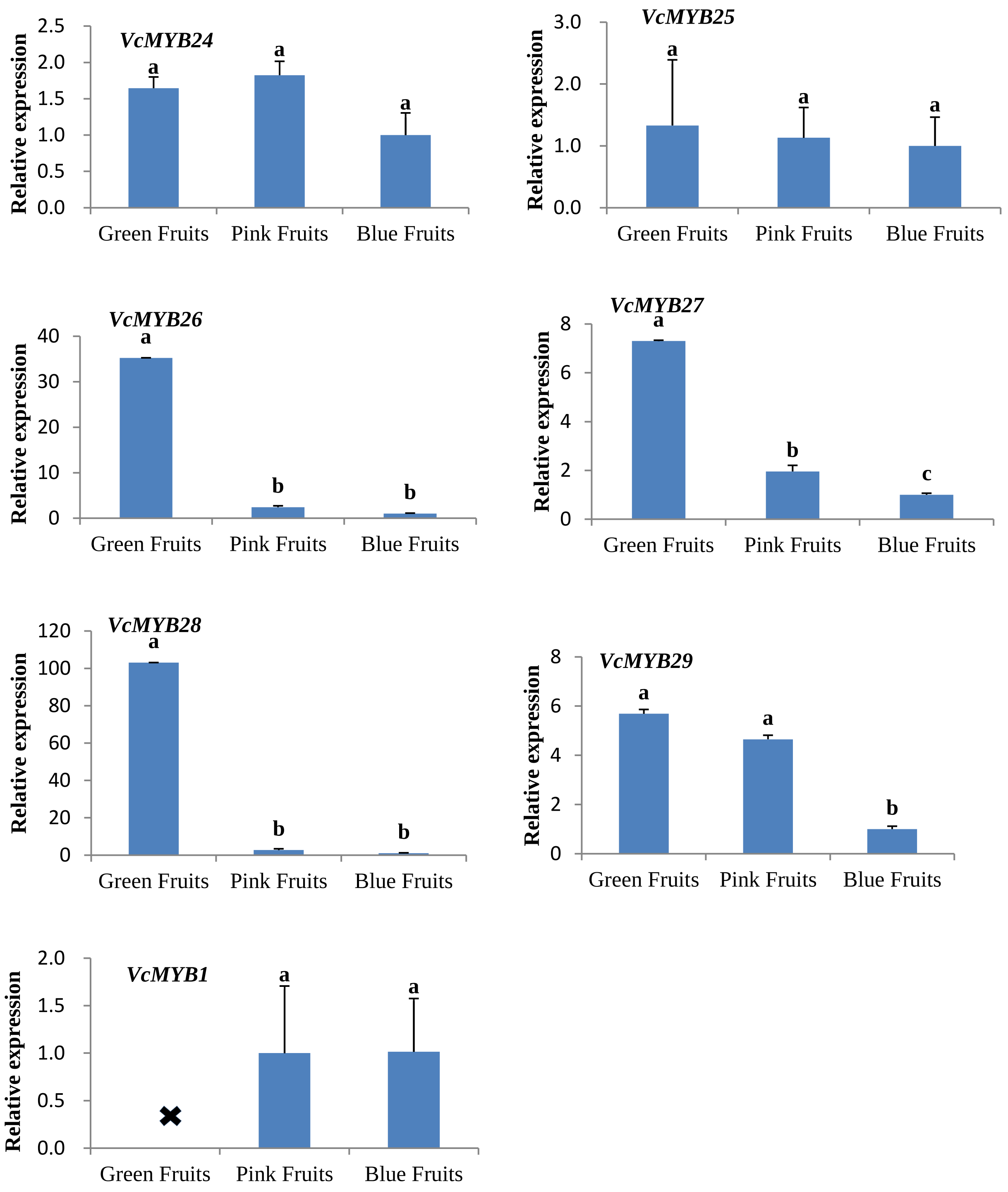

Supplementary Figure 3.Transcript abundance of MYB genes at different developmental stages in blueberries. Transcript nine MYB genes at different developmental stages in blueberries. All values were calculated based on the geometric mean of the housekeeping genes, *VcGADPH* and *VcSAND*, at each stage. Different English letters represents that the one-way ANOVA difference was significant(P<0.05). The mean ± SE of biological triplicates were taken for every value.

1.2 Supplementary Table

Supplementary Table S1. Statistics of sequences length from Illumina and SMRT sequencing data.

Sample	No. of Unigene/FLNC	No. of Unigene/FLNC			Average Unigene/FLCN length(bp)	N50	N90
		>=1 kb	>=2 kb	>=3 kb			
Illumina	104,068	18,849	7,042	2,476	677	1131	265
SMRT	42,740	42,646	24,312	9,869	2452	2754	1621

Supplementary Table S2. FPKM values and annotation of flavonoid-synthesis genes in blueberries.

Name	Gene ID	Green fruits-1 (fpkm)	Green fruits-2 (fpkm)	Pink fruits-1 (fpkm)	Pink fruits-2 (fpkm)	Blue fruits-1 (fpkm)	Blue fruits-2 (fpkm)	Function	GenBank (gi)/Swissprot (tr)
VcPAL	c4817/f8p1/2531	57.66	61.8	0.77	1.11	0.74	0.37	phenylalanine ammonia-lyase	gi|720021891
VcC4H	c37948/f2p6/1796	154.25	148.37	201.17	223.57	305.65	157.59	cinnamate 4-hydroxylase	gi|927092641
Vc4CL	c6503/f2p4/1871	24.48	29.88	2.35	2.16	3.04	3.22	4-coumarate--CoA ligase	tr|O24146
VcCHS	c41364/f1p1/2875	5.68	4.29	20.16	29.88	22.63	14.61	chalcone synthase	gi|606214816
VcCHI	c67730.graph_c0	156.14	204.75	158.66	247.88	187.13	123.01	chalcone isomerase	gi|726969188
VcF3H	c13490/f4p0/1416	49.62	53.86	265.24	477.58	269.19	236.59	flavanone 3-hydroxylase	gi|219944305
VcF3'H	c10242/f1p4/1668	15.32	19.46	26.72	39.47	33.88	21.11	flavonoid 3'-hydroxylase	gi|606214812
VcF3'5'H	c896/f12p29/1800	0.49	0.86	1145.01	1384.87	1215.49	959.96	flavonoid 3',5'-hydroxylase	gi|164454800
VcDFR	c6548/f1p2/1382	155.31	159.79	151.5	198.67	161.83	119.84	dihydroflavonol 4-reductase	gi|588295057
VcANS	c1528/f7p3/1389	180.98	230.16	651.75	722.09	827.14	590.28	leucoanthocyanidin dioxygenase	gi|403406452
VcUFGT	c1081/f13p4/1864	11.88	15.89	103.46	105.14	103.65	90.66	UDP-glucose: flavonoid 3-O-glucosyltransferase	gi|606214808
VcCCoAOMT	c17329/f1p0/1152	6.6	7.73	209.46	181.59	259.77	132.31	caffeoyl-CoA O-methyltransferase	gi|658004994
VcFLS	c28862/f1p0/1273	26.33	44.7	0.93	2.07	0.3	0.85	flavonol synthase	gi|827523408
VcANR	c9059/f1p1/1675	2.01	3.31	0	0	0	0	anthocyanidin reductase	gi|403406436

VcLAR	c15348/f1p3/1620	0.98	1.9	0	0.74	0.05	0	leucoanthocyanidin reductase	gi|403406448
VcMYB1	c83830.graph_c0	15.51	30.72	91.61	90.63	84.67	52.58	Transcription factor MYB114 (Arabidopsis thalian)	tr|Q9FNV8
VcMYB22	c6582/f2p0/1470	6.42	5.67	3.31	8.57	3.79	5.63	Myb-related protein	tr|P81392
VcMYB23	c27452/f1p0/1364	36.42	30.62	0	0.06	0.42	0.21	Transcription factor MYB86	tr|Q8LPH6
VcMYB24	c1736/f5p0/1833	2.97	3.57	2.9	1.35	2.28	1.91	myb-related protein	gi|720064986
VcMYB25	c12778/f2p1/1471	109.49	73.88	91.5	73.02	75.03	68.12	Transcription factor MYB44	tr|Q9FDW1
VcMYB26	c22453/f1p0/1417	4.28	4.45	0.17	0.26	0.06	0.06	Transcription factor MYB44	tr|Q9FDW1
VcMYB27	c1233/f3p2/3618	1.43	2.13	0.23	0.22	0.17	0.85	myb-related protein	gi|359476374
VcMYB28	c16957/f1p0/1555	1.38	2.13	0	0	0	0	Transcription factor MYB98	tr|Q9S7L2
VcMYB29	c1686/f6p3/2709	17.54	17.04	3.62	4.99	2.66	5.55	Myb-related protein	tr|Q9S7G7

Supplementary Table S3. List of primers used in this study.

Gene name	Primer pairs (5'-3' Forward/Reverse)
**VcPAL-RT-PCR**	CTCCGACAAGTCCTGGTGGAGCATGCG/CGACCGACATTCCTTTATCCTGTTTCC
**VcC4H-RT-PCR**	GGCATCACTATCGGGCGTTTGGTCC/CATTTCCTCCCCAACCCAACTTCAG
**Vc4CL-RT-PCR**	CACCGATACAATCCCTAAAGCTCC/CATACATAAATTTCCCCGTCTCTCC
**VcCHS-RT-PCR**	CCGCGGCCCAAACCATTCTCCCGG/CCGGGGTGAGCGATCCAGAAGATTG
**VcCHI-RT-PCR**	GGAATCCAAGATCAAGGTCGAGAATGCG/CATTCAAACCAAAAGCTGTTGGGCAAAC
**VcF3H-RT-PCR**	GACGAGCCAATCACGTTCATGGAG/CATCCCCGAAACTGTATCAACAGCC
**VcF3'H-RT-PCR**	GGTCCAGCTACTAACCGCGACATTGG/CACAGCCGTGTTACTCATATACAAAC
**VcF3'5'H-RT-PCR**	CAAGCCTTATGCAAAGAAGCCTTTC/CATAAACCTTTCCGGATTGAACTCC
**VcDFR-RT-PCR**	GTCCCCACTGAGTTTAAGGGGATTCC/CGTTCCATTTCCATTTCCATTGGCAGTTG
**VcANS-RT-PCR**	GAGGAGCTCACCAGCATCGGCAAC/GTCCATCAGCTCTTCCGGGACGCCG
**VcUFGT-RT-PCR**	AGATAGCGGCATTAGCT/CAACCGCAGTGGTTTATG
**VcCCoAOMT-RT-PCR**	CTCAACAAAATGCTAGCAGCTGATCC/CAGTTTACCTGTTTATTTTTTAACCC
**VcFLS-RT-PCR**	GTCGTGGCCGGTGTTCTTAGAACCACC/GCTAAGAAAGTTCACTAGGGTCTGGTCC
**VcANR-RT-PCR**	CTTTGAAGATTTCCCTTCCAAAG/GACACACAGTGGTATATTAGAAAAGG
**VcLAR-RT-PCR**	CCTGATGAAGTTGAAGTGTGCGAAC/GCAATTCCATTGGCGGTTACGCC
**VcMYB22-RT-PCR**	CTCATTTGAAGAAGCAGCTCAAG/CAGAGGGCTTTTTTTGGCC
**VcMYB23-RT-PCR**	CACCCATAAAGCGATAAGCG/CTCTCCCTCCACCTTCATAG
**VcMYB24-RT-PCR**	CGGTTTACCCATATACCCCG/GAACTAGGACAGGGTGGTTGCG
**VcMYB25-RT-PCR**	CACGCTCAAGAGGAAGTGCTCG/CTCGAATCGCTGACGTCAGATC
**VcMYB26-RT-PCR**	CCAATTCCAAACTGTGTGGTGG/GTCTGGAATTCCACGAATCC

**VcMYB27-RT-PCR**	GATGATATCACTGCGGAAAAC/GTAATGTGCAACATTCTAGAATAC
**VcMYB1-RT-PCR**	GGTGGGATGACATGTTGTTCGATTATG/GGTCCACATTGTCCATAAAAATATTAC
**VcMYB28-RT-PCR**	CGCAAATGCAAGTACAACTCC/CACAGCCTTACTATTGTGATC
**VcMYB29-RT-PCR**	GCAAGAAGAGAGCAAAACATG/GAGATGGGTCCTCCTCATCTTC
**VcGAPDH-RT-PCR**	GGTTATCAATGATAGGTTTGGCA/CAGTCCTTGCTTGATGGACC
**VcSAND-RT-PCR**	AAGCATCTCTTCATCCTGATGA/GATTGTATCTTGGCAGGCAA
**AtUBQ10-RT-PCR**	CGTTAAGACGTTGACTGGGAAAACT/ GCTTTCACGTTATCAATGGTGTCA
**VcMYB1-pRI101**	ggaattccatatgATGATTCAATTAAAGGGTGC/cgcggatccTTACAGTACTGCTTGTTCATC
**VcMYB1-pGreen62-SK**	cgcggatccATGATTCAATTAAAGGGTGC/ccgctcgagTTACAGTACTGCTTGTTCATC
**VcMYB1-pGADT7**	ccggaattcATGATTCAATTAAAGGGTGC/cgcggatccTTACAGTACTGCTTGTTCATC
**VcMYB1-pET32a**	cgcggatccATGATTCAATTAAAGGGTGC/ccggaattcTTACAGTACTGCTTGTTCATC
**proVcDFR-pAbAi-P**	cgagctcCAAATGTTTAATTGAATTATTTAAAT/acgcgtcgacTATCAAATAAAACCCGATATAC
**proVcDFR-pAbAi-P1**	cgagctcACAACCTTATAATTGCGATGAC/acgcgtcgacTATCAAATAAAACCCGATATAC
**proVcDFR-pAbAi-P2**	cgagctcTGGACAAGACCGTTCAATCTC/acgcgtcgacTGACACACGCGCACGTGCTCAC
**proVcDFR-pAbAi-P3**	cgagctcGAGAGACCAAATGGATAGTTG/acgcgtcgacTGACACACGCGCACGTGCTCAC
**proVcDFR-pAbAi-P4**	cgagctcCAAATGTTTAATTGAATTATTTAAAT/acgcgtcgacTCAGGACTGTCTGATGGAGCC
**proVcDFR-pGreen0800-Luc**	ccccctcgaggtcgaCAAATGTTTAATTGAATTATTT/tagaactagtggatcGACTGGTTCGAATATCAA
**proVcDFR-pCAMBIA1301-GUS**	acgaattcgagctcggtaccCAAATGTTTAATTGAATTATTT/tcagatctaccatggGACTGGTTCGAATATCAA
**proVcDFR-SP1-Genome walk**	GGCAACTCCAATAGGTGTTTC
**proVcDFR-SP2-Genome walk**	CTTCAAATTGCCTGGATCGCG
**proVcDFR-SP3-Genome walk**	CGGTTGCTCGAACAACATAGC

Supplementary Table S4. Comparison of flavonoid synthesis-related genes in blueberries.

Gene Name	Illumina sequence	SMRT sequence	Hybrid sequence	Li et al. 2016	Sun et al. 2015	Li et al.2012
PAL	6	25	25	13	11	8
C4H	5	11	12	7	4	3
4CL	26	37	54	11	13	8
CHS	8	12	14	13	2	9
CHI	4	0	4	4	3	4
F3H	2	5	6	9	6	4
F3'H	2	6	6	×	6	5
F3'5'H	5	14	17	×	7	3
DFR	8	10	14	5	20	7
ANS	2	4	4	1	4	5

UFGT	33	54	68	73	105	23
CCoAOMT	4	16	16	×	8	0
FLS	6	2	6	×	3	8
ANR	2	3	3	×	2	1
LAR	3	5	6	×	5	4
